# Naturalistic fNIRS assessment reveals decline in executive function and altered prefrontal activation following social media use in college students

**DOI:** 10.1038/s41598-025-20844-7

**Published:** 2025-10-22

**Authors:** Anna Aitken, Ali Rahimpour Jounghani, Laura Moreno Carbonell, Daniel Tadeo, Anupam Kumar, Seth Crawford, Audrey K. Bowden, S. M. Hadi Hosseini

**Affiliations:** 1 Computational Brain Research and Intervention (C-BRAIN) Laboratory, Department of Psychiatry and Behavioral Sciences, School of Medicine, Stanford University, Stanford, CA USA; 2 Department of Bioengineering, Stanford University, Stanford, CA USA; 3 Department of Biomedical Engineering, Vanderbilt University, Nashville, TN USA

**Keywords:** Social media, fNIRS, Executive functioning, Emotion, College students, Neuroscience, Psychology

## Abstract

As of 2023, 69% of adults and 81% of teens in the U.S. use social media regularly. This study investigates the immediate impact of social media consumption on executive functioning (EF) and emotion in college students using a wearable, naturalistic functional near-infrared spectroscopy (fNIRS) system. Twenty participants completed EF tasks and self-report questionnaires both before and after a brief social media exposure. Over half (55%) met criteria for social media addiction, with an average Instagram usage of five hours per week. Behaviorally, social media exposure led to reduced accuracy in EF tasks, including n-back and Go/No-Go paradigms. Neurally, increased medial prefrontal cortex (mPFC) activation suggested greater cognitive effort and performance monitoring, while decreased dorsolateral (dlPFC) and ventrolateral prefrontal cortex (vlPFC) activation reflected impairments in working memory and inhibition. Reduced inferior frontal gyrus (IFG) activity was also linked to difficulties suppressing motor responses. Emotional changes were modest, with the control group reporting slightly lower happiness post-intervention. These findings demonstrate the cognitive cost of social media use and underscore the potential of wearable fNIRS for real-world brain monitoring. This approach may inform future interventions and support precision mental health strategies targeting digital behavior.

**Supplementary Information:**

The online version contains supplementary material available at 10.1038/s41598-025-20844-7.

## Introduction

As of 2023, social media usage is widespread, with 69% of adults and 81% of teens in the U.S. actively engaging with these platforms^1^. A 2023 survey revealed that U.S. teenagers spend an average of 4.8 h per day on social media platforms, while only 10.5% of teenagers report spending one hour or less on these platforms^2^. Many individuals exhibit addictive behaviors towards social media akin to a smoker’s unconscious habit of reaching for a cigarette^3^. Adolescents and young adults, in particular, often display a muscle memory reflex that prompts them to pick up their phones and open a social media app.

While the academic discourse frequently delves into how prolonged social media use impacts adolescent mental health and academic performance, this study shifts the focus to examining the immediate effects of social media use on brain activity and behavior. The surge in global social media usage among college and university students has sparked discussions about its potential influence on academic performance. However, existing evidence on this connection remains inconclusive. A review conducted by Valkenburg et al.^4^ revealed varying perspectives, with some researchers characterizing the association between mental health issues and social media usage as ‘weak’ or 'inconsistent,' while others deemed it ‘substantial’ and ‘deleterious’^4^. Suárez-Perdomo’s study in 2022 identified significant differences in procrastination behaviors linked to social network addiction among students, yet no notable variations were found in academic performance^5^. These disparities underscore the complexities in the literature surrounding the impact of prolonged social media use on different facets of well-being. While social media use has been linked to cognitive challenges, it also serves as a tool for emotion regulation and social connection—particularly during periods of stress or isolation, such as the COVID-19 pandemic. When used in moderation, social media may offer psychological benefits that complement, rather than hinder, well-being^6^.

Executive function (EF) encompasses higher-level cognitive skills that coordinate and control cognitive abilities and behaviors, including inhibition, working memory, and cognitive flexibility. First, inhibitory control involves overriding automatic responses, allowing individuals to change their reactions consciously^7,8^. Second, working memory, essential for reasoning and understanding language, requires individuals to actively hold and manipulate information^7^. Third, cognitive flexibility, emerging later in development, involves shifting perspectives and adapting to new situations^7,9^. EFs are critical for academic success, influencing areas such as problem-solving, learning, planning, and impulse inhibition^7,9–12^, but they also play a pivotal role in daily life tasks such as time management, decision-making, and regulating emotions. Impairments in EF can reduce attention span, decrease cognitive abilities, and lower overall productivity^13^. It is also important to consider the temporal nature of social media effects—while platforms may support emotion regulation in the long run, their short-term impact may disrupt immediate cognitive performance, especially when used during tasks such as studying or homework.

Previous neuroimaging studies using tools like EEG and fMRI have provided valuable insights into executive functioning (EF)^14^. However, their application is often constrained by the need for controlled environments and static setups, limiting their ability to assess EF during everyday activities. Our study aims to address this gap by leveraging a wearable functional near-infrared spectroscopy (fNIRS) device^15^. This portable and user-friendly platform allows for the real-time, standardized assessment of EF during naturalistic settings. Unlike traditional approaches, our wearable fNIRS system enables the exploration of neurocognitive processes as they unfold in real-world scenarios, offering an ecologically valid perspective on how individuals manage cognitive demands in their daily routines.

The relationship between specific aspects of EF and distinct regions of the prefrontal cortex (PFC) is well-established. These PFC areas are intricately connected to various brain regions, including other cortical areas and structures associated with emotional reactivity, attention control, and stress response^9,16^. While EFs are primarily mediated by prefrontal cortical function, they are also subject to modulation by neurotransmission inputs of dopamine, noradrenaline, serotonin, and acetylcholine. The adaptability of cognitive behavior in response to environmental changes is facilitated by these neurotransmitter systems’ modulating effects on executive function. However, alterations in these systems can significantly impact EF^9,11^.

Dopamine, a neurotransmitter with roles extending beyond EF, is implicated in processes like reward encoding and drug addiction. The relationship between dopamine and prefrontal EF follows an inverted U-shaped curve, whereby both low and excessive dopamine levels can impair cognitive performance^11^. Optimal prefrontal function is supported by moderate dopamine levels, which enhance performance in tasks involving attention and cognitive flexibility. Norepinephrine activity influences all four cognitive processes supporting executive function. Active in both the medial PFC (mPFC) and orbitofrontal cortex (OFC), it regulates overall arousal levels and establishes basal levels of cortical activity. Consequently, arousal levels dictate the activation of various norepinephrine receptor types, contributing to the modulation of cognitive processes. The serotonin system plays a crucial role in the OFC mediating response inhibition. Lower serotonin levels are associated with impaired response inhibition, highlighting the multifaceted influence of neurotransmitter systems on executive function. Finally, the inferior frontal cortex also plays an important role in response inhibition. The right inferior frontal gyrus (IFG) is considered a key node for the inhibition of motor responses, which is a core aspect of impulse control^11,17^.

Emerging evidence suggests that social media use may transiently alter neurotransmitter activity, particularly dopamine, due to its rewarding and reinforcing nature. Given the critical role of dopamine, norepinephrine, and serotonin in supporting executive function, such alterations may partially explain the immediate effects of social media on EF. These mechanisms highlight a potential link between the neurochemical changes induced by social media and the observed impairments in cognitive processes like attention and response inhibition.

A recent study used fNIRS to examine the impact of tablet use on preschoolers’ executive function in lab environment. The device revealed distinctive patterns in the PFC between heavy tablet users and non-users^18^. Among the 30 tablet users, 16 were classified as *heavy users* based on their daily screen time exceeding the mean level (M = 17.98 min, SD = 14.29), uncontrolled or unlimited tablet use, and engagement in multiple activities on the tablet. The remaining 14 users, classified as *low users*, were excluded from the comparative analysis. The study incorporated an EF measure, specifically the Dimensional Change Card Sort (DCCS) task, which assesses cognitive flexibility, concurrently with the fNIRS tool.

The results revealed that *heavy users* exhibited lower performance on the DCCS task compared to *non-tablet users*, indicating better EF performance among the latter group. Moreover, the activation patterns in the prefrontal cortex varied significantly between the two groups. Interestingly, the pattern observed in the 'non-user’ group aligned with expectations and was considered typical. In contrast, the pattern observed in the 'heavy-user’ group was unexpected. The heavy-user pattern was characterized by a significant decrease in oxygenated hemoglobin concentration (Oxy-Hb) and an increase in deoxygenated hemoglobin concentration (deOxy-Hb) in the PFC. Finally, this exemplifies the feasibility of using fNIRS to explore the neural underpinnings of EF in the context of modern technology use, such as social media engagement in lab environment.

We aimed to pioneer the use of our wearable fNIRS system^19^ to examine the immediate effects of social media consumption and its implications for EF and emotion. The low-cost, portability, and ecological validity of our wearable fNIRS platform are highly valuable and enable research to be done regardless of location or expense. This approach represents a novel contribution to the existing body of research as it explores real-time neurocognitive and emotional responses to social media use, particularly among the demographic most immersed in these platforms.

Our study aims to investigate how social media consumption affects PFC activity during cognitive tasks, using our wearable fNIRS system in a natural setting. By "natural setting," we refer to an environment that closely resembles everyday conditions rather than a traditional, controlled laboratory. In this case, all data were collected in a quiet, private room in a student residence building, which allowed participants to engage in social media use in a context that mirrors typical, real-world interactions with technology. Notably, the social media interaction in this study was passive, involving only scrolling without active engagement such as commenting or messaging. This approach enhances ecological validity by capturing brain activity in settings that participants are more accustomed to, providing a more accurate reflection of how social media impacts cognitive functioning during daily life. These real-world settings are where many college students engage with social media—often in between or during academic tasks—raising concerns that even brief periods of passive scrolling may interfere with executive function during cognitively demanding periods.

Our approach provides the flexibility to monitor brain activity while participants engage in everyday behaviors. In this study, we explored the impact of social media use on EF and emotional state by examining behavioral performance and correlating it with prefrontal cortical activity. Participants were divided into two groups: a control group and a social media use group. Both groups completed EF tasks, such as working memory and inhibition exercises, at two sessions—before and after engaging in their respective activities.

Using fNIRS, we measured prefrontal activity via oxygenated hemoglobin levels. Based on our hypothesis, we anticipated a group-by-session interaction, where participants in the social media use group would show increased prefrontal activity in regions associated with EF, such as working memory and inhibition, relative to the control group. These results would provide insight into the neural correlates of social media’s impact on cognitive and emotional processing. Furthermore, we predict that social media use would result in a decline in positive emotions and impairments in executive function. The findings of this study offer valuable insights into the influence of social media on cognitive processes and its potential implications for cognitive health, particularly in relation to EF.

## Results

Table 1 summarizes descriptive statistics and sociodemographic information for the social media and control groups. Social media use was assessed via time spent on Instagram and SMAS scores. Sociodemographic variables include age, sex, race, and ethnicity. Between-group comparisons were conducted using appropriate statistical tests. Participants classified as “addicted” were defined based on the SMAS threshold.

**Table 1:** Participant Characteristics and Social Media Use Measures.

Measure	Social Media (N = 10)	Control (N = 10)	Total Sample (N = 20)	*p*-value
Age, mean (SD)	21.75 (1.23)	21.81 (1.11)	21.78 (1.15)	*p* = 0.77
Sex, female, n (%)	5 (50%)	6 (60%)	11 (55%)	*p* = 0.90
*Race*				
Asian	0 (0%)	2 (20%)	2 (10%)	
Black/African American	3 (30%)	3 (30%)	6 (30%)	
White	7 (70%)	5 (50%)	12 (60%)	
Ethnicity, Latino/Hispanic	5 (50%)	5 (50%)	10 (50%)	p = 1.0
Time spent on Instagram (hrs/week), mean (SD)	7.8 (5.2)	2.2 (1.3)	4.99 (4.4)	-
SMAS sum score, mean (SD)	102.4 (15.6)	83.1 (17.2)	92.65 (20.2)	-
Participants classified as addicted, n (%)	8 (80%)	3 (30%)	11 (55%)	-

Participants were asked to report their weekly Instagram usage and complete a questionnaire regarding their relationship to social media. The Social Media Addiction Scale (SMAS) results revealed that 55% of participants are classified as addicted to social media (threshold for classification of addiction was a score of > 90). It also revealed that participants on average used Instagram for 5 h/week, although this did not account for screen time on other social media platforms.

Our study included 20 participants, divided into a control group (${n}_{Control group}$:10) and a social media use group (${n}_{Social Media group}$:10). Both groups completed EF tasks, such as working memory and inhibition exercises, at two sessions—before and after the intervention.

### Behavioral findings

We investigate whether the EF task measurements showed differences in behavioral performances on the EF tasks between groups before and after the intervention (more info in the Methods section). Descriptive statistics (means and standard deviations) for all outcome measures are summarized in Table S1, separated by group (social media vs. control) and condition (pre- and post-intervention), to provide a comprehensive overview of group-level trends. The experimental, social media group viewed their Instagram following page, whereas the control group were exposed to black and white nature photos, which are more neutral and emotionally uncharged.

**Fig. 1:**
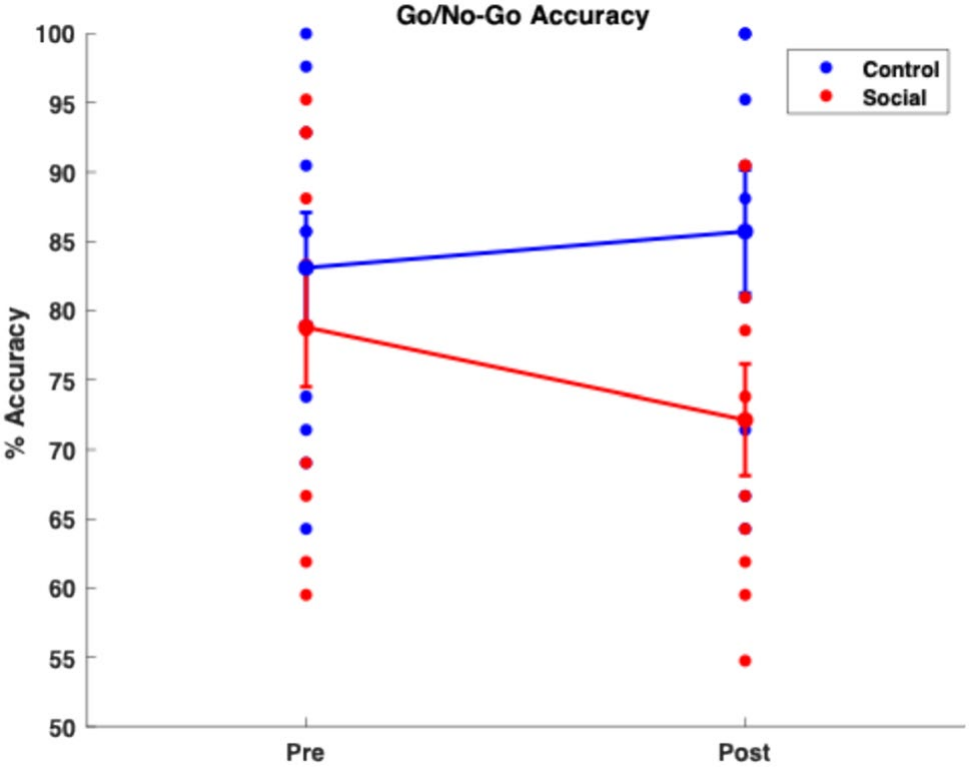
Significant group × session interaction in Go/No-Go correct rejection rate (p = 0.0017). Participants in the social media group showed reduced inhibitory performance post-exposure, while the control group exhibited improved performance.

As shown in Fig. 1, the social media group demonstrated significantly poorer change scores in Go/No-Go correct rejection performance compared to the control group (Mann–Whitney U = 6.125, $${\text{p}}_{\text{adjusted}}=0.012,$$ Rank-Biserial Correlation, $$r=-0.66),$$ despite exhibiting similar hit rate reaction times (Fig. S1).

This finding supports the hypothesis that executive function task performance declines following social media use. Additionally, on 2-back + 3-back vs. 0-back task contrast, the social media group showed no statistically significant difference in change scores compared to the control group after social media use (${\text{p}}_{\text{adjusted}}=0.17$, Fig. S2). However, a large negative effect size (Rank-Biserial Correlation, $r=-0.55$) suggests a potentially meaningful practical difference in change memory scores between groups.

The results of the Discrete Emotions Questionnaire (DEQ) revealed no significant changes in emotional states following social media use, except for a difference observed in the Happiness Items Score. The analysis revealed no statistically significant group-by-session interaction effect on the Happiness Items of the DEQ, assessed using a Mann–Whitney U test on change scores (${\text{p}}_{\text{adjusted}}=0.1163$)*;* moreover, a medium negative effect size (Rank-Biserial Correlation, $\text{r}=-0.2667$) indicated a practical difference in change scores between groups (Fig. S3).

### fNIRS findings

**Fig. 2:**
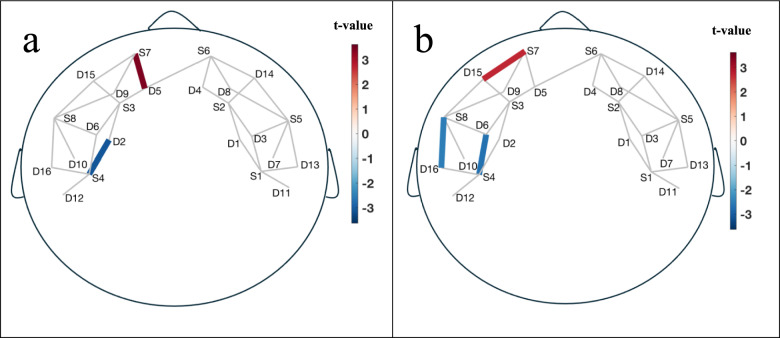
Channel maps of group and session interaction effect showing significant cortical Oxy-Hb activation in the left prefrontal areas during (**a**) 2-back + 3-back vs. 0-back and (**b**) No-Go vs. Go. The color bar represents t-value at those locations.

Significant interaction effect between group and session were found in the fNIRS activation patterns during two EF tasks (n-back and Go/No-Go tasks). Figure 2 shows the left-lateralized dorsolateral prefrontal cortex (dlPFC), mPFC, ventrolateral prefrontal cortex (vlPFC), IFG activation. During the 2-back + 3-back vs. 0-back task contrast (Fig. 2a), increased activity in the mPFC $(t_{S7 - D5} = 3.32,p < 0.05,Cohen^{\prime}s\;d = 1.1)$; Refer to Fig. 5b for the corresponding source-detector pairs layout) is often related to the monitoring of ongoing performance and high regulation of cognitive effort in social media usage comparing to control group^20^. During the Go/No-Go task (No-Go vs. Go), the mPFC $(t_{S7 - D15} = 3.13,p < 0.05,Cohen^{\prime}s\;d = 1.4)$ is involved in monitoring actions and detecting errors (Fig. 2b)^21^. Increased mPFC activity is associated with high effort to inhibit responses and performance monitoring influenced by social media^21^.

The dlPFC is crucial for maintaining and manipulating information in working memory^22–24^. Decreased activity in the dlPFC $(t_{S4 - D2 } = - 3.3,p < 0.05,Cohen^{\prime}s\;d = 0.8)$ during the n-back task reflects less cognitive load to update and maintain the working memory representations. The dlPFC is also engaged in maintaining task rules and exerting top-down control to inhibit inappropriate responses^23,24^. Decreased activity in the dlPFC $(t_{S4 - D6} = - 2.7,p < 0.05,\;Cohen^{\prime}s d = 0.9)$ during the Go/No-Go task is associated with the unsuccessful inhibition of responses to No-Go stimuli affected by social media ^24^.

The vlPFC/IFG is involved in response inhibition and conflict resolution^25–28^. Decreased activity in the vlPFC $(t_{S8 - D16} = - 2.5,p < 0.05,Cohen^{\prime}s\;d = 1.3)$ during the Go/No-Go task reflects low cognitive effort to inhibit responses and a failure to resolve conflicts between Go and No-Go signals^27^. The vlPFC/IFG is also critically involved in inhibitory control^25–27^. Decreased activity in the IFG during the Go/No-Go task is strongly associated with the failure to suppress motor responses and withhold actions in response to No-Go stimuli^28^ resulted from social media.

To further explore task-specific effects, we conducted additional analyses separating the n-back conditions and reporting all pairwise contrasts (see Supplementary Figs. S4 and S5). These revealed that the 2-back condition elicited significant Oxy-Hb activation in the left IFG ($t_{S8 - D16} = 3.11,p < 0.05,Cohen^{\prime}s\;d = 0.37$), while the 3-back condition showed activation in the left superior frontal gyrus and bilateral mPFC ($t_{S2 - D4, \, S3 - D2, S3 - D5\;combined} = 2.14,p < 0.05,Cohen^{\prime}s\;d = 0.23)$. Compared to 0-back, 2-back showed increased IFG activation $(t_{S8 - D6,\;S8 - D9\;combined} = 2.55,\;p < 0.05, Cohen^{\prime}s = 0.78)$ and dlPFC deactivation $(t_{S4 - D2} = - 2.35,p < 0.05,Cohen^{\prime}s\;d = 0.67)$, while 3-back showed increased left superior frontal activation $(t_{S3 - D2,\;S3 - D5\;combined} = 2.28,\;p < 0.05,\;Cohen^{\prime}s \;d = 0.83)$. Furthermore, the 3-back vs. 2-back contrast revealed increased activation in the right mPFC $(t_{S6 - D4} = 2.75, p < 0.05, Cohen^{\prime}s d = 0.82)$. No significant activation was found in the 0-back condition alone. Similarly, no significant activation was observed in the go and no-go trials when analyzed separately. No significant group × session interaction effects were found for any of the above contrasts.

## Discussion

As of 2023, an estimated 6.92 billion people—approximately 86% of the global population—use smartphones, with platforms like Instagram, Twitter, TikTok, and Facebook dominating digital engagement^29^. Our study contributes to a growing body of literature examining the complex relationship between social media use and executive functioning (EF). Using a wearable, portable fNIRS system in a naturalistic setting, we provide novel insights into how social media consumption affects both cognitive function and emotional states in college students—not only in academic contexts but also in everyday behaviors such as social interaction, time management, and decision-making.

In our study, social media engagement was quantified using weekly Instagram usage (mean = 5 h) and standardized addiction criteria, with 55% of participants classified as addicted based on the Bergen Social Media Addiction Scale. Specifically, participants in the social media group exhibited lower accuracy than those in the control group. These findings suggest that even brief passive social media use may impair cognitive functions such as inhibitory control and working memory, though further analyses are needed to determine the scope and specificity of these effects. Notably, our study focused solely on passive use—participants scrolled their Instagram feed without interacting or posting.

Our fNIRS data provided a more detailed view of these impairments by highlighting specific changes in brain activity. We observed increased mPFC activity during n-back and Go/No-Go tasks, which is typically associated with heightened cognitive effort and performance monitoring^20,21^ while social media usage. This suggests that participants may have exerted more effort to maintain their performance on these tasks after using social media, possibly as a compensatory response to the cognitive demands imposed by social media engagement.

Conversely, decreased activity resulting from social media use was observed in the dlPFC, vlPFC and IFG, which are regions critical for maintaining working memory and exerting response inhibition, respectively. The reduction in activity in these areas indicates a diminished capacity to effectively manage working memory and inhibit inappropriate responses, reinforcing the idea that social media use can disrupt normal EF^23–28^.

Although emotional changes in our study were modest, the distinction between emotion and emotion regulation is important. Social media is often used to downregulate stress or boost mood^30,31^, yet our data suggest that this regulatory intent might incur a cognitive cost, particularly during concurrent task demands. The observed increase in mPFC activity, for example, may reflect a dual role in both performance monitoring and emotional self-regulation, underscoring the intertwined nature of executive and affective processes in naturalistic settings.

fNIRS findings in this study were predominantly left-lateralized, consistent with the left prefrontal cortex’s dominant role in verbal working memory, language-related executive processes, and cognitive control—as supported by prior neuroimaging literature^32,33^. This aligns with the nature of the tasks used in our study, which involved visually presented letters in both the n-back and Go/No-Go paradigms. Previous research has shown that such verbal stimuli further engage left-lateralized prefrontal regions involved in symbolic processing and active manipulation of information^34,35^. This supports the hypothesis that left-hemisphere regions are particularly recruited during tasks requiring active maintenance and manipulation of information.

Interestingly, while the Go/No-Go task showed clear group differences in behavioral performance, the N-back task did not, despite notable differences in brain activation. This discrepancy suggests that social media exposure may impact specific executive functions—such as inhibitory control—more readily than working memory at the behavioral level. The increased mPFC activation and decreased dlPFC activation during the N-back task may reflect greater neural effort required by the social media group to maintain comparable performance, possibly indicating compensatory recruitment. These findings underscore the importance of integrating behavioral and neural data, as neural changes may precede or underlie subtle behavioral effects not captured by standard accuracy metrics.

These findings align with a growing literature on the negative impact of media multitasking on cognitive abilities, including attention and memory^36,37^. However, our study advances the field by being the first to measure these effects using fNIRS in a naturalistic setting, offering a level of ecological validity often lacking in laboratory-based studies.

Overall, our findings highlight potential—but not definitive—effects of social media on EF. Prior studies suggest that strong executive function may serve as a protective buffer against addictive behaviors, whereas impaired inhibitory control may reinforce problematic social media use^38^. Yet, the literature also shows inconsistencies, particularly in academic settings: some studies report no change—or even improvements—in EF following exposure to certain emotionally engaging social media content^6^. Our results align with Lench et al.^39^, who found that emotional states such as happiness can influence cognition^39^. It is also possible that participants found the control condition less engaging, reducing reported happiness. These inconsistencies highlight the importance of content type, individual differences in emotional regulation, and usage context in shaping outcomes.

The implications of these findings are broad, spanning cognitive health, education, and social development. First, a decline in EF may impair individuals’ ability to plan, focus, and make effective decisions, with downstream effects on mental well-being^40^. Secondly, these impairments also have direct implications for academic and professional outcomes. Executive functions underpin learning, problem-solving, and task management. Diminished EF following social media use may negatively affect academic achievement, reduce productivity, and limit long-term career development—potentially influencing both personal and societal progress^41^. Moreover, the social consequences of diminished EF are equally important. Skills such as empathy, impulse control, and perspective-taking are essential for successful social interaction^42^. A decline in these domains may impair emotional regulation, hinder communication, and degrade relationship quality—especially among youth immersed in social media^43,44^.

These findings underscore the need for targeted interventions at both policy and educational levels. Policymakers could promote healthy online behavior by encouraging balanced digital use and integrating digital literacy into curricula. Programs should also emphasize cognitive self-regulation, critical thinking, and awareness of the cognitive risks associated with excessive social media use. Moreover, educators and parents play a crucial role in shaping mindful digital habits. Raising awareness about the cognitive impact of social media and fostering environments that support balanced engagement may help reduce risk and improve outcomes.

While our study offers key insights, several limitations must be acknowledged. The small sample size limits statistical power and generalizability. Although the fNIRS system effectively captured cortical activity, it does not measure deeper brain structures involved in EF. Future research should replicate these findings in larger, more diverse populations and explore the long-term cognitive and emotional effects of social media use. Additionally, context matters: many participants experienced high school or early college during the COVID-19 pandemic, when social media served as a primary tool for connection and emotional regulation. These historical factors likely shaped both behavior and brain responses.

Another limitation is the lack of standardized assessments for mood, well-being, or neuropsychiatric symptoms. Although participants reported no diagnoses, undetected conditions such as stress, anxiety, or depression could have influenced performance. Future studies should include validated tools to assess emotional state and mental health, particularly given the life-stage transitions common in college populations.

We also did not assess for undiagnosed attention-related conditions, such as ADHD or ADD, which could affect EF. Including screening measures in future studies would strengthen group comparisons. However, random assignment likely distributed these traits evenly across groups, minimizing confounding effects.

While we explored correlations between behavioral performance and fNIRS activity in mPFC, dlPFC, IFG, and vlPFC, no significant associations emerged. This may reflect limited statistical power, inter-individual variability in cognitive strategies, or non-linear relationships between neural activity and behavior. Larger samples and more sensitive analytic approaches will be essential to clarify these relationships.

Programs like the Truman Platform^45^ and TestDrive^46^ provide controlled environments for promoting healthy digital behavior. Research from ACM SIGCHI has further explored how design features and feedback can support self-regulation in online spaces^47^. However, these approaches are not yet widely adopted. Our findings support the need to scale such interventions and evaluate their impact across diverse settings.

Future research should also focus on developing and evaluating new tools—such as digital detox programs, cognitive self-regulation techniques, or neurofeedback interventions^48^—to help mitigate the cognitive costs of social media use. Examining how different content types (e.g., visual vs. text-based, passive vs. interactive) affect executive function and emotion regulation will further illuminate how digital engagement shapes mental health over time.

## Conclusion

This study demonstrates that even brief exposure to social media can alter executive functioning in college students, affecting key cognitive processes like working memory and response inhibition. Using a wearable fNIRS system in a naturalistic setting, we observed increased mPFC activity, suggesting heightened cognitive effort and performance monitoring. In contrast, reduced activation in dlPFC and vlPFC reflected diminished working memory capacity and inhibitory control. Notably, decreased activation in IFG—a region essential for suppressing inappropriate motor responses—was associated with poorer No-Go task performance.

Our use of portable neuroimaging underscores the potential for at-home, real-world brain monitoring, offering a scalable tool for advancing precision mental health and understanding how digital habits impact cognitive wellness in daily life.

## Methods

### Participants

Participants were gathered via a recruitment flyer posted across campus. A pool of 20 undergraduate students (11 women and 9 men; mean age ± SD = 20.75 ± 1.164) were randomly recruited (See Table 1). All experimental sessions were conducted between February 9th and 25th 2024. Participants in the social media group accessed their personal Instagram accounts using version 322.1 of the mobile application. Participants were then randomly assigned to either a control group (i.e., who scrolling through neutral photos on social media) or experimental group (i.e., who scrolling through social media). None of the participants reported any neurological disorder or injury that would prevent them from performing executive functioning tasks. This study was approved by the Stanford University School of Medicine Institutional Review Board for research ethics and human participants. In addition, informed consent was obtained from all participants for participation in the study and publication of any identifying information or images in an online open-access publication. All identifying details have been excluded from the submitted materials.

All methods were performed in accordance with relevant guidelines and regulations, as approved by the Stanford University School of Medicine Institutional Review Board for research ethics and human participants. In addition, informed consent was obtained from all participants for participation in the study and publication of any identifying information or images in an online open-access publication. All identifying details have been excluded from the submitted materials.

### Experimental procedures

**Fig. 3:**
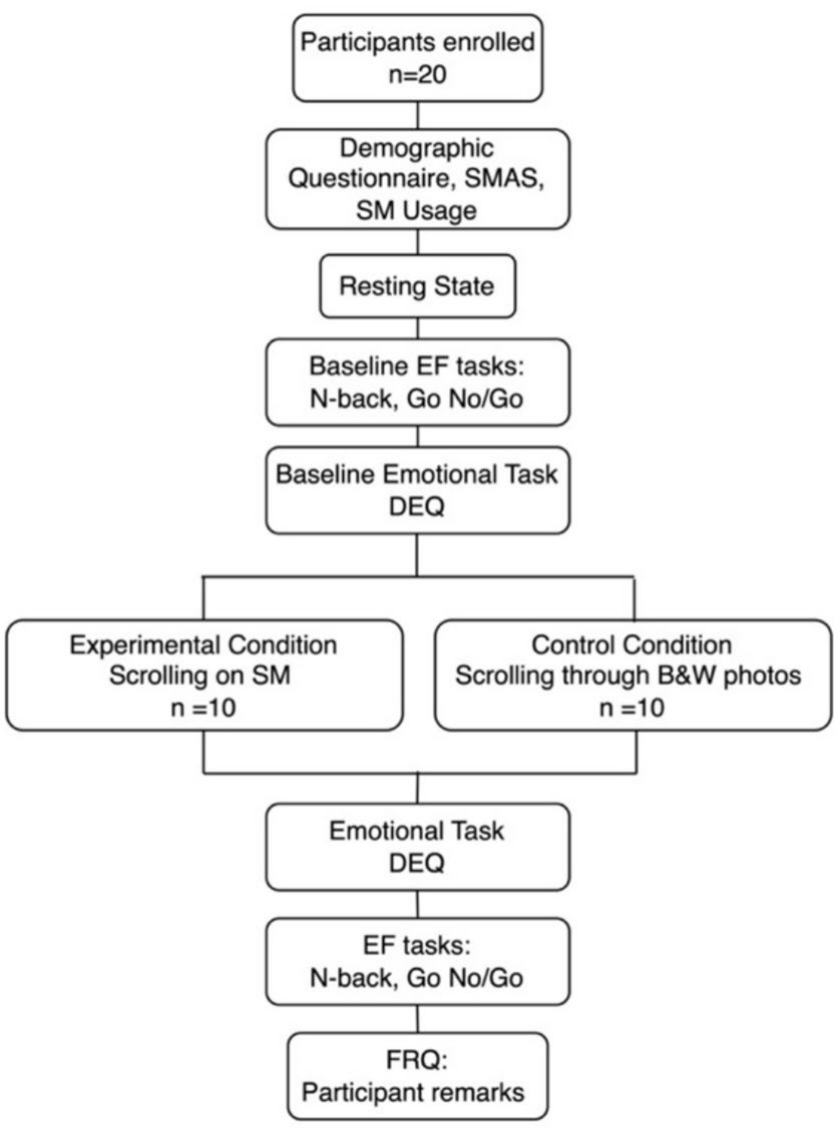
Procedure flow chart.

The detailed procedure is presented in Fig. 3. Data collection was completed in one 80-min session after participants completed a consent form and were re-screened for exclusion criteria. Upon arrival, participants filled out basic demographic information and completed the SMAS and a questionnaire about general social media consumption (e.g., screen time, app type). They then put on the wearable fNIRS headband and recorded the basal brain activity at rest, establishing a baseline reading, before conducting the first round of EF tasks and emotion tasks.

The resting state condition established the baseline brain activity specific to the person. Participants were instructed to close their eyes and relax for 7 min. Following this, emotional and EF tasks were performed to establish a baseline performance before the intervention (social media use) or control condition. After this, the participant scrolled on their Instagram “following” page for 15 min. Participants used their personal Instagram accounts during this phase. They were instructed to refrain from interacting with any content—specifically, they were not permitted to like, comment, message, or post. This ensured consistent passive engagement across participants. Importantly, this interaction was passive in nature, involving only scrolling through content without engaging in active behaviors such as liking, commenting, or messaging. This design choice was intentional to isolate the neural and cognitive effects of passive content consumption. A 15-min session allowed time for collecting enough fNIRS data, while still mimicking a typical social media session.

Following this, the same assessments were performed post-social media use to compare results with the baseline. The control group underwent the same procedure but scrolled through a curated selection of black-and-white nature images with captions on a single, neutral Instagram account that was preloaded with nature-themed photos and silent videos, mimicking a typical feed layout while minimizing emotional salience and interactivity. Importantly, no audio stimuli were presented in either the experimental or control condition; all media were either static images or silent videos to avoid auditory confounds.

### Outcome measures and experimental procedure

This study employs a mix of objective and subjective measures. The objective measures include standardized, validated computer-based tasks targeting various facets of EF, such as working memory and inhibition. Subjective measures involve self-report and informant-report questionnaires designed to evaluate emotion regulation in participants’ daily life situations. Stimuli for both groups included static images and short silent videos, and no audio content was presented in either condition to avoid auditory confounds.

### Objective measures

**Fig. 4:**
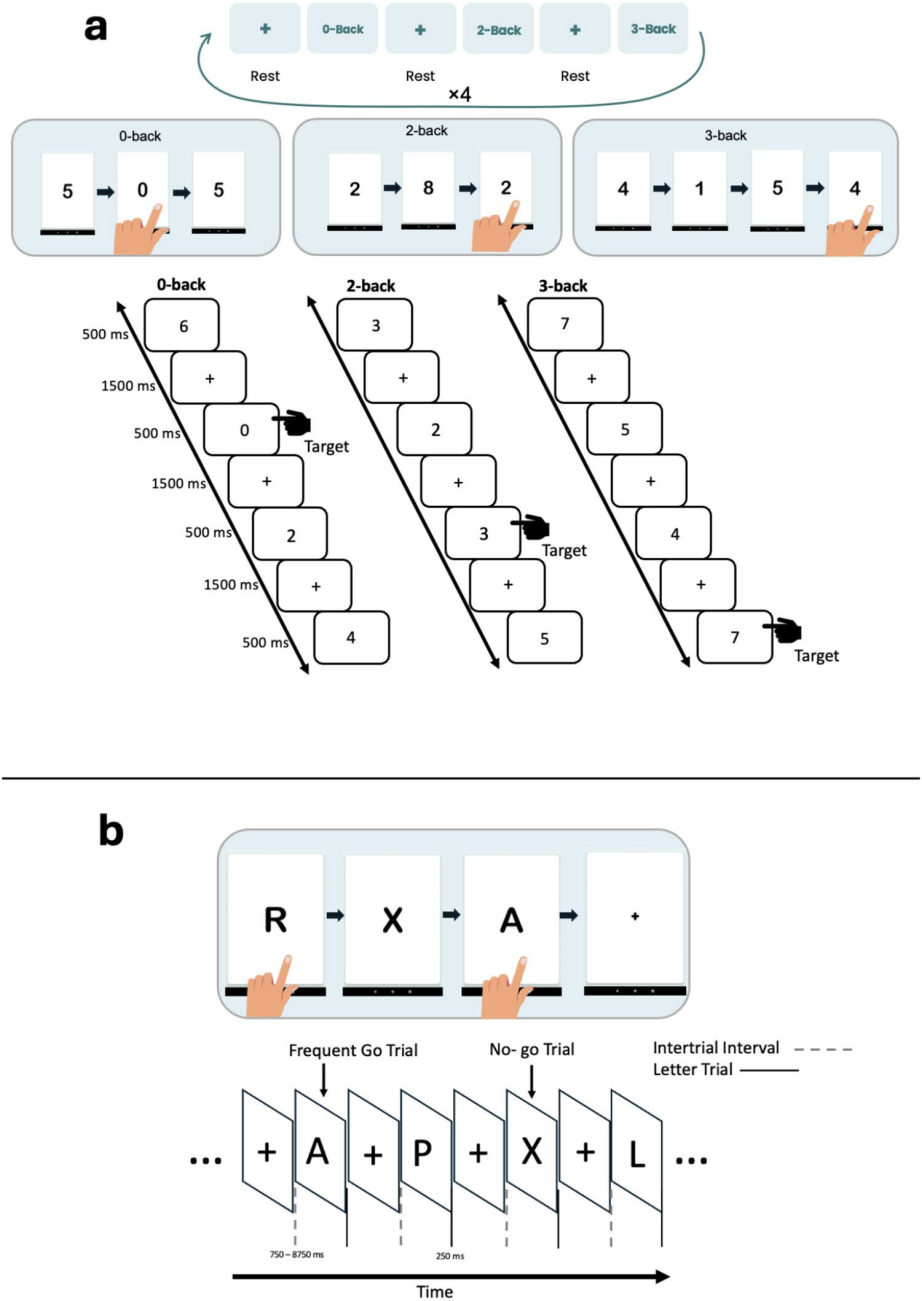
Schematic representations of the cognitive tasks implemented in the tablet application: (**a**) the *n-back* working memory task and (**b**) the *Go/No-Go* response inhibition task. Each panel includes the task design and visual layout as presented to participants.

*N-back Task* This task measures working memory. Participants monitored a series of numeric stimuli (digits 0–9) and indicated whether the current number matched the one presented *n* steps back in the sequence (Fig. 4a). The task included three levels: 0-back, 2-back, and 3-back, each repeated four times, resulting in 12 blocks. Each block contained 20 trials, with target (match) stimuli presented on approximately 30% of trials. Stimuli were shown for 500 ms, followed by an inter-stimulus interval of 1500 ms (fixation cross).

Participants responded by tapping the screen when a target was detected. Accuracy was calculated as the proportion of correct responses to targets (hits) relative to total target trials in each block, and reaction time was measured for correct hits. The difficulty of the task increased with higher *n* levels, placing greater demands on working memory updating and maintenance.

*Go/No-Go Task* This task (Fig. 4b) assessed inhibitory control using letter stimuli. Of all trials containing stimuli (168 trials), 75% were Go trials (letters other than X), and 25% were No-Go trials (letter X). These stimulus trials were interleaved with jittered fixation intervals (750–2500 ms; mean = 1750 ms) to reduce anticipatory responses and enhance temporal unpredictability. Only trials with stimulus presentation were used in accuracy and reaction time analyses.

*Instructions to participants "In this game, you will see letters appear one at a time on screen. Tap the screen for every letter except X. Press as quickly and correctly as you can."*

*Accuracy scoring* Go trial accuracy reflected the proportion of correct responses (hits), indicating omission errors when missed. No-Go trial accuracy reflected correctly withheld responses (correct rejections), capturing commission errors.

### Subjective measures

*Discrete Emotions Questionnaire (DEQ)* The DEQ is sensitive to eight distinct state emotions: anger, disgust, fear, anxiety, sadness, happiness, relaxation, and desire. Participants answer questions like: “While [doing this action], to what extent did you feel [emotion]?” They rank their experience from 1 (not at all) to 7 (an extreme amount). The outcome measure is the sum of the scores indicated in each of the eight categories. The DEQ was selected due to its established use in studies assessing emotional responses to short-term experimental stimuli^49^. While it focuses on discrete, highly valenced and arousal-based emotions (e.g., anger, happiness, anxiety), it provides a practical and validated framework for capturing emotional shifts in brief interventions such as ours. Future research may consider incorporating additional tools that assess a broader spectrum of affective states, including lower-arousal or more nuanced emotional experiences.

*Social Media Addiction Scale (SMAS)* The SMAS measures dependence on social media. It reflects six features of addiction proposed in Griffiths’s components model of addiction: salience, mood modification, tolerance, withdrawal, conflict, and relapse. Participants rank how they relate to statements from 1 (very rarely) to 5 (very often). The outcome measure is the sum of the scores of all questions.

### Neuroimaging

**Fig. 5:**
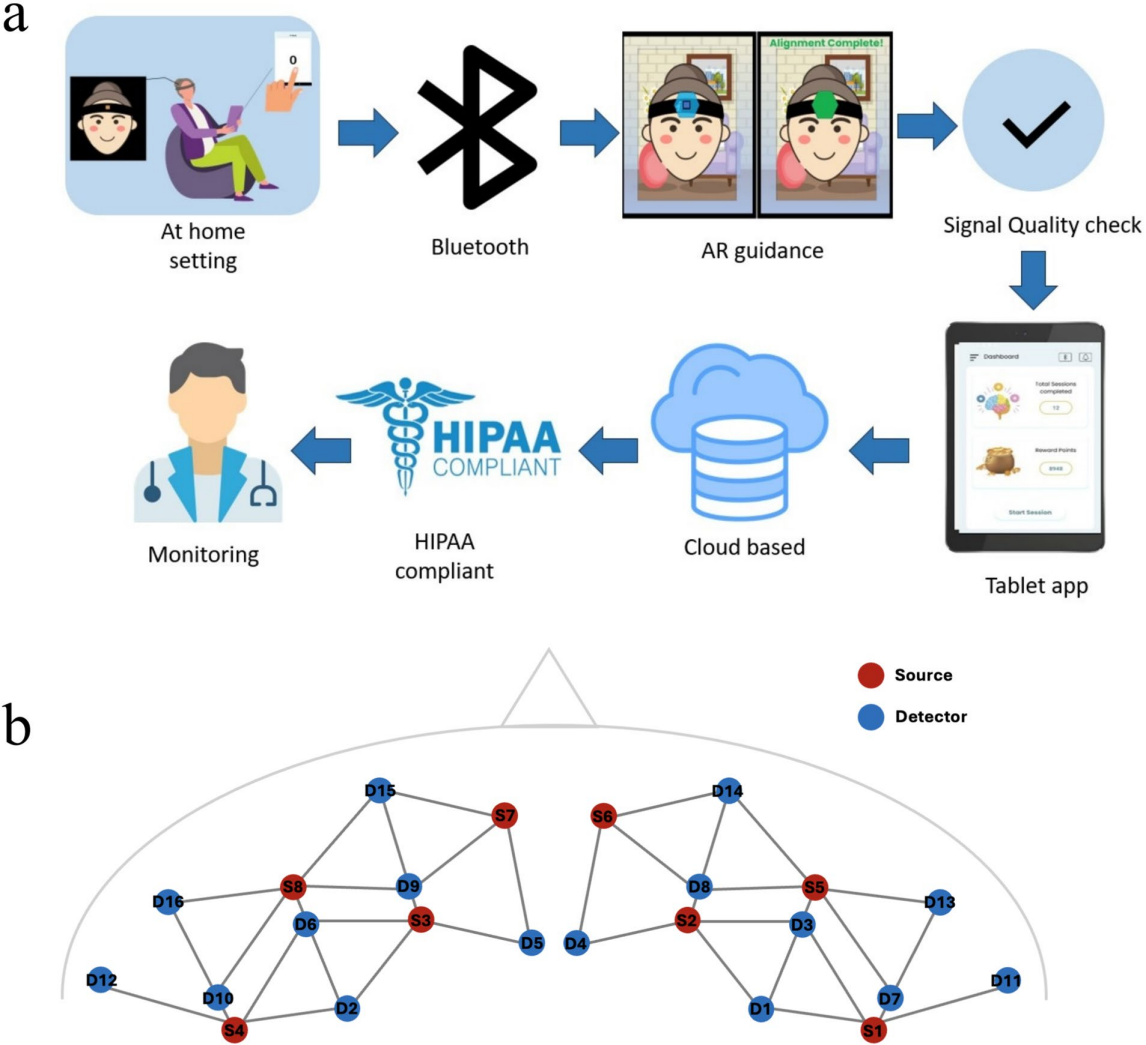
(**a**) Wearable fNIRS platform ^48^ for measuring brain activity in natural setting (See participant example in Figure S6) and (**b**) corresponding source-detector pairs layout.

The study used a recently validated, custom-built fNIRS platform^19,50^, developed collaboratively by the Bowden Biomedical Optics Laboratory (BBOL) at Vanderbilt and Stanford University C-BRAIN Laboratory. This wearable, wireless, multi-channel, smartphone-operated optical imaging system was specifically designed to monitor prefrontal activity in naturalistic settings (Fig. 5).

The device consisted of 7 light sources and 16 detectors, configured to form 33 regular and 4 short channels, covering bilateral prefrontal regions, including 4 short channels for superficial signal correction. Channels were mapped to key cortical areas (dlPFC, vlPFC, IFG, mPFC) based on standard 10–20 system positioning and MNI coordinate references described in Table S2. The system operated at two wavelengths (740 nm and 850 nm) and had a sampling rate of 7 Hz. Data were collected using a wireless, battery-powered setup and transmitted to a tablet for acquisition and real-time quality monitoring.

### Data analysis

The preliminary phase of analyzing fNIRS data included a rigorous quality control procedure to pinpoint and eliminate channels of inferior quality. Channels with a Signal Noise Ratio (SNR) and Quality Index (QI) below set thresholds (SNR < 20 dB and QI < 0.5) were discarded, resulting in an average exclusion rate of approximately 8% of channels across participants. Baseline corrections were made to the raw intensity signals to address issues like direct current shifts, concatenation effects, and global signals^51^. Motion artifacts were tackled using wavelet filtering^52^ with a sym8 wavelet, discarding data points that deviated by more than five standard deviations from the mean, primarily focusing on motion and very low-frequency disturbances. The raw data were then used to extract levels of Oxy-Hb and deOxy-Hb using the modified Beer-Lambert law.

The AR-IRLS regression model by Santosa et al.^54^, implemented via the BrainAnalyzIR toolbox^53,54^, improved signal fidelity and identified regressors by utilizing data from short channels, reducing physiological and additional motion-related artifacts and enhancing fNIRS measurement reliability^55^.

A general linear mixed regression model was used for further statistical analysis to investigate the influence of different experimental tasks (N-Back and Go/No-Go tasks) on cortical activation. The dependent variables represented by beta (β) corresponded to the effects for Oxy-Hb and deOxy-Hb under each condition. We also performed post hoc contrast analyses between conditions within each task to further explore specific activation patterns^48^. The Benjamini–Hochberg correction method based on the False Discovery Rate (FDR) was applied to reduce the risk of false positives.

Behavioral measures were analyzed using nonparametric statistical tests to account for the small sample size and the non-normal distribution of the data. Mann–Whitney U tests were applied for within-group comparisons (pre- vs. post-intervention) and also for between-group comparisons of change scores. Multiple comparisons were corrected using the Holm–Bonferroni method to control the family-wise error rate. All analyses were conducted in MATLAB R2022b.

## Supplementary Information

Supplementary Information.

## Data Availability

The data that support the findings of this study are available from the corresponding author, A.RJ, upon request (email: rahimpur@stanford.edu)

## Code availability

The code that supports the findings of this study is available from the corresponding author, A.RJ, upon request (email: rahimpur@stanford.edu).
