# Supplementary material for: Naturalistic fNIRS assessment reveals decline in executive function and altered prefrontal activation following social media use in college students
Source: Sci Rep. 2025 Oct 22;15:36960. doi: 10.1038/s41598-025-20844-7 (PMC12546605; doi:10.1038/s41598-025-20844-7)

**Naturalistic fNIRS Assessment Reveals Decline in Executive Function and Altered Prefrontal Activation Following Social Media Use in College Students**

***Supplementary Materials***

**Table S1. Descriptive statistics for behavioral, emotional, and fNIRS outcome measures.**

| Measures | Group | Pre-mean (SD) | Post-mean (SD) | Change score mean (SD) |
| --- | --- | --- | --- | --- |
| Go/No-Go accuracy |  |  |  |  |
| Correct Rejection rate | Social Media | 78.3 $\pm$ 3.55 | 72.2 $\pm$ 4.15 | -6.1$\pm$ 3.88 |
|  | Control | 83.8 $\pm$ 3.3 | 85.6 $\pm$ 5.5 | 1.8 $\pm$ 2.66 |
| Hit rate | Social Media | 90.8 $\pm$ 5.2 | 92.8.6 $\pm7$.6 | 2.06 $\pm$ 2.4 |
|  | Control | 94.7 $\pm$ 4.4 | 90.6 $\pm$ 6.9 | -4.1 $\pm$ 2.5 |
| N-Back accuracy |  |  |  |  |
| 0-back | Social Media | 96 $\pm$ 3.4 | 94 $\pm$ 3.8 | -2 $\pm$ 0.5 |
|  | Control | 95 $\pm$ 4.3 | 96 $\pm$ 1.2 | 1 $\pm$ 2.3 |
| 2-back | Social Media | 88 $\pm$ 2.1 | 82 $\pm$ 4.6 | -6 $\pm$ 2.5^*^ |
|  | Control | 88 $\pm$ 4.2 | 92 $\pm$ 1.6 | 4 $\pm$ 2.8 |
| 3-back | Social Media | 80.4 $\pm$ 5.5 | 72.8 $\pm$ 6.6 | -7.6 $\pm$ 1.3^*^ |
|  | Control | 82.6 $\pm$ 3.9 | 90.4 $\pm$ 2.5 | 7.8 $\pm$ 1.4 |
| DEQ Happiness | Social Media | 4.33 $\pm$ 0.26 | 4.37 $\pm$ 0.31 | 0.04 $\pm$ 0.1 |
|  | Control | 4.82 $\pm$ 0.04 | 3.92 $\pm$ 0.35 | 0.8 $\pm$ 0.31 |
| N-Back fNIRS activation |  |  |  |  |
| mPFC | Social Media | 0.55 $\pm$ 0.9 | 3.1 $\pm$ 0.82 | 2.55 $\pm$ 0.77^*^ |
|  | Control | -2.7 $\pm$ 1.1 | -1.2 $\pm$ 0.4 | -0.5 $\pm$ 0.9 |
| dlPFC | Social Media | -0.3 $\pm$ 1.2 | -2.7 $\pm$ 0.8 | -2.4 $\pm$ 1.1^*^ |
|  | Control | 0.7 $\pm$ 0.7 | 1.6 $\pm$ 0.33 | 0.9 $\pm$ 0.44 |
| vlPFC/IFG | Social Media | 2.3 $\pm$ 1.3 | 2.15 $\pm$ 0.91 | -0.15 $\pm$ 0.63 |
|  | Control | 1.6 $\pm$ 0.72 | 1.92 $\pm$ 1.3 | 0.32 $\pm$ 0.58 |
| Go/No-Go fNIRS activation |  |  |  |  |
| mPFC | Social Media | 2.22 $\pm$ 1.5 | 1.93 $\pm$ 1.7 | -0.29 $\pm$1.3 |
|  | Control | 2.8 $\pm$ 0.82 | 1.4 $\pm$ 1.2 | 1.4 $\pm$ 0.88^*^ |
| dlPFC | Social Media | 4.43 $\pm$ 1.9 | 4.04 $\pm$ 1.7 | -0.39 $\pm$ 1.5 |
|  | Control | 3.01 $\pm$ 1.4 | 3.42 $\pm$ 2.1 | 0.41 $\pm$ 1.8 |
| vlPFC/IFG | Social Media | -2.5 $\pm$ 1.6 | -3.02 $\pm$ 1.3 | -0.52 $\pm$ 1.1 |
|  | Control | -2.02 $\pm$ 1.1 | -2.9 $\pm$ 1.8 | -0.88 $\pm$ 1.5 |

Note: Asterisks (*) *indicate statistically significant differences in change score mean (p < 0.05).*

**Figure S1.** Group by session interaction effect in Go/No-Go hit rate reaction time

**
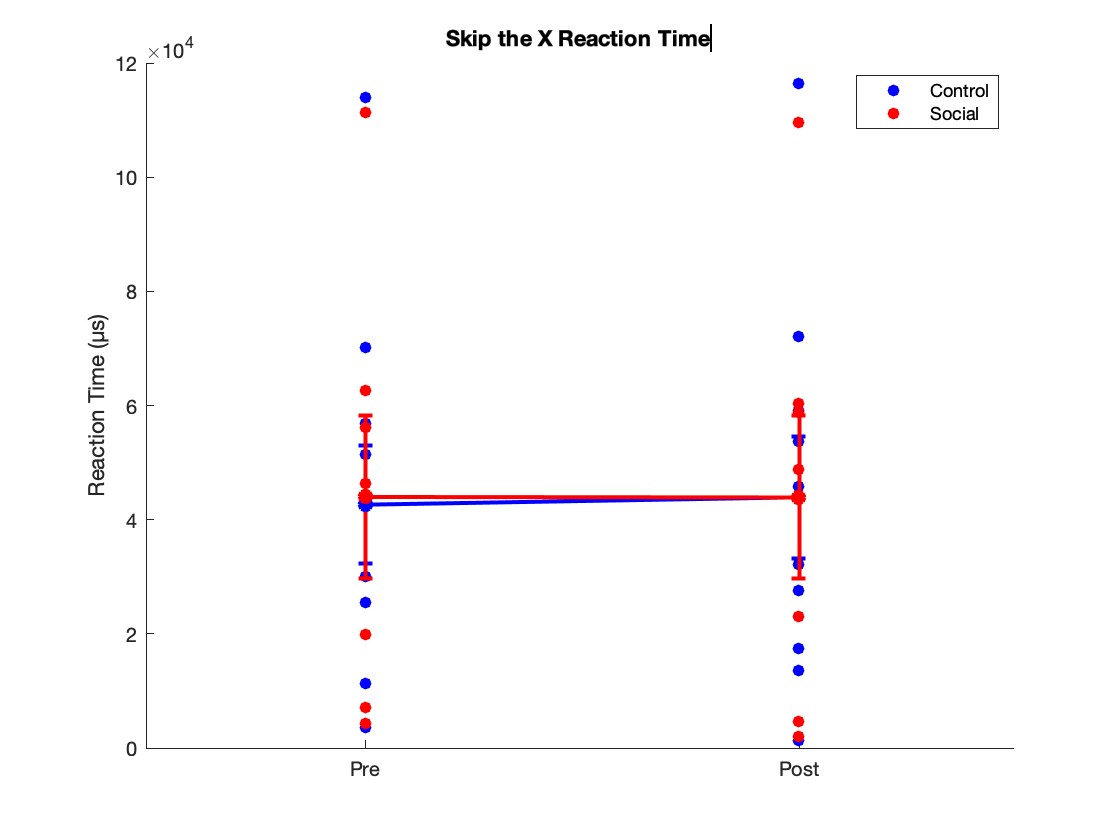
**

**Figure S2.** Interaction effect of group by session on behavioral accuracy for the average of 2-back + 3-back

**
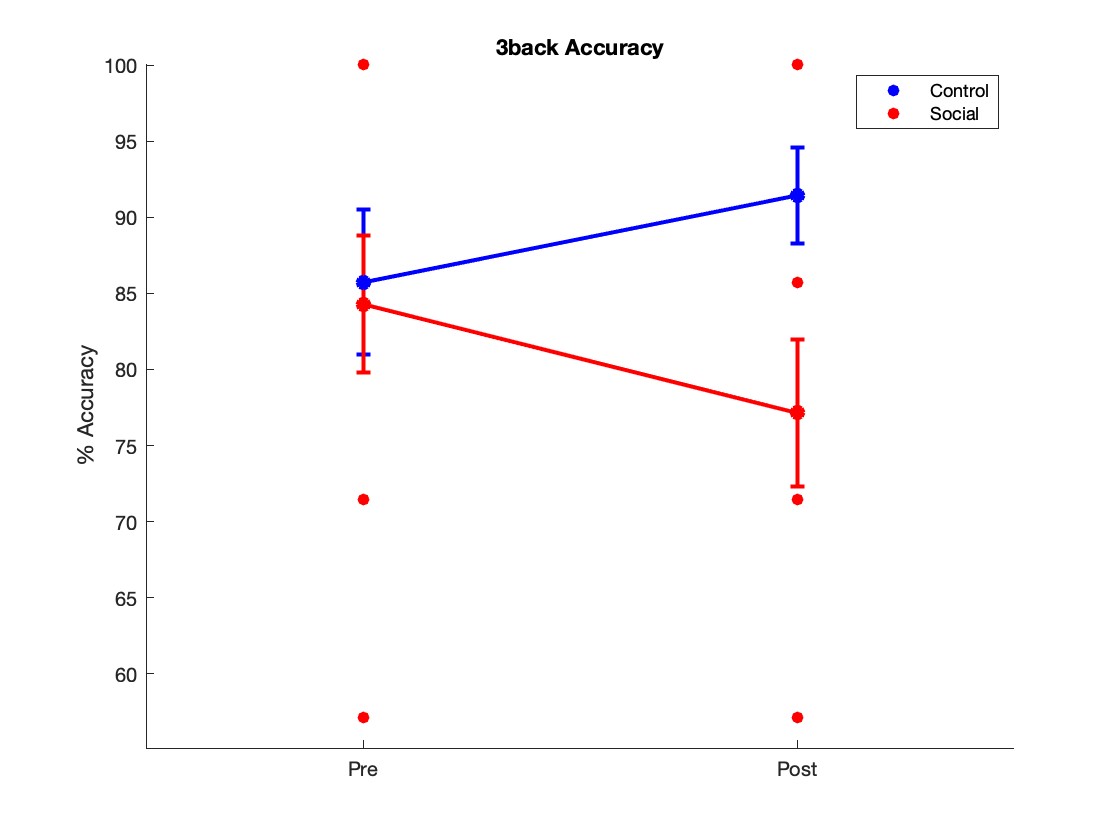
**

**Figure S3.** Group by session significant interaction effect in average DEQ Happiness item score****

**Figure S4.** Channel maps of significant post–pre cortical activation for Oxy-Hb and deOxy-Hb in the prefrontal cortex during the n-back task conditions: (a) 0-back, (b) 2-back, and (c) 3-back. Contrast maps are also shown: (d) 2-back vs. 0-back, (e) 3-back vs. 0-back, and (f) 3-back vs. 2-back. The color bar represents the t-values at each channel location across all participants.

| **deOxy-Hb** | **Oxy-Hb** |
| --- | --- |
| **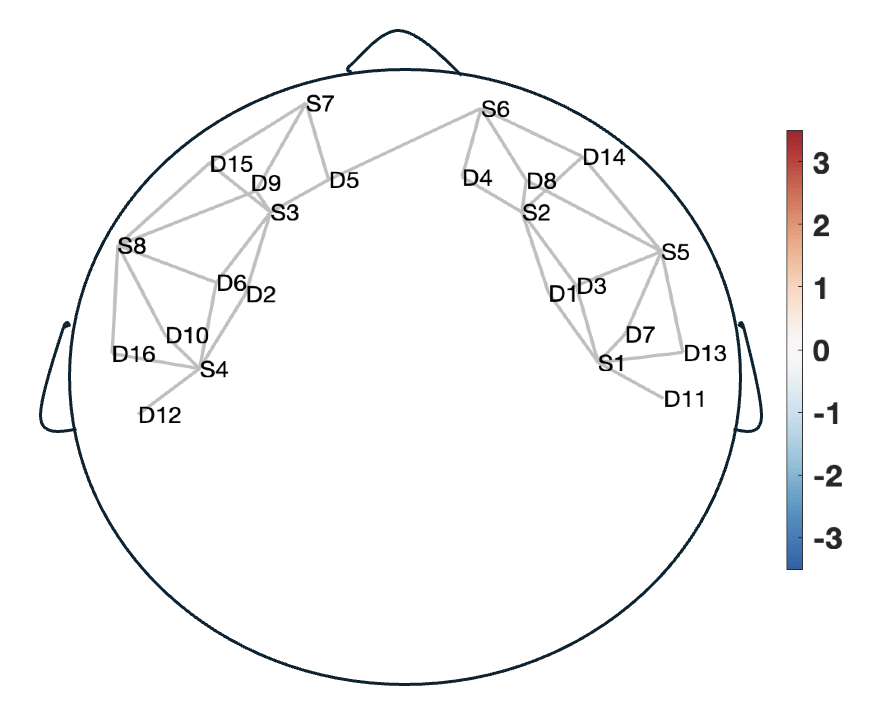**  a  **t-value**  **t-value** | 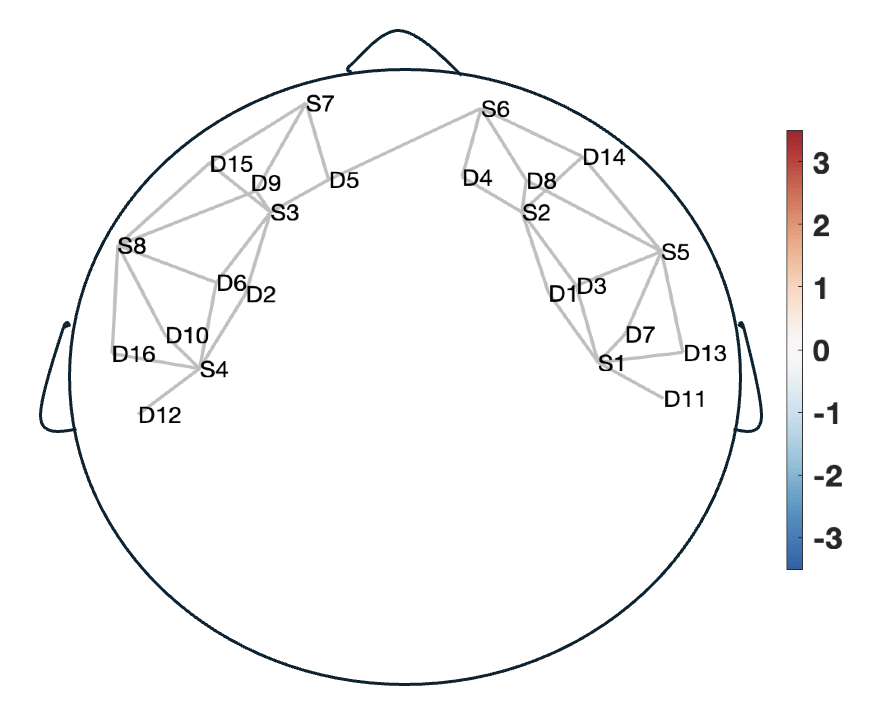  **t-value** |
| **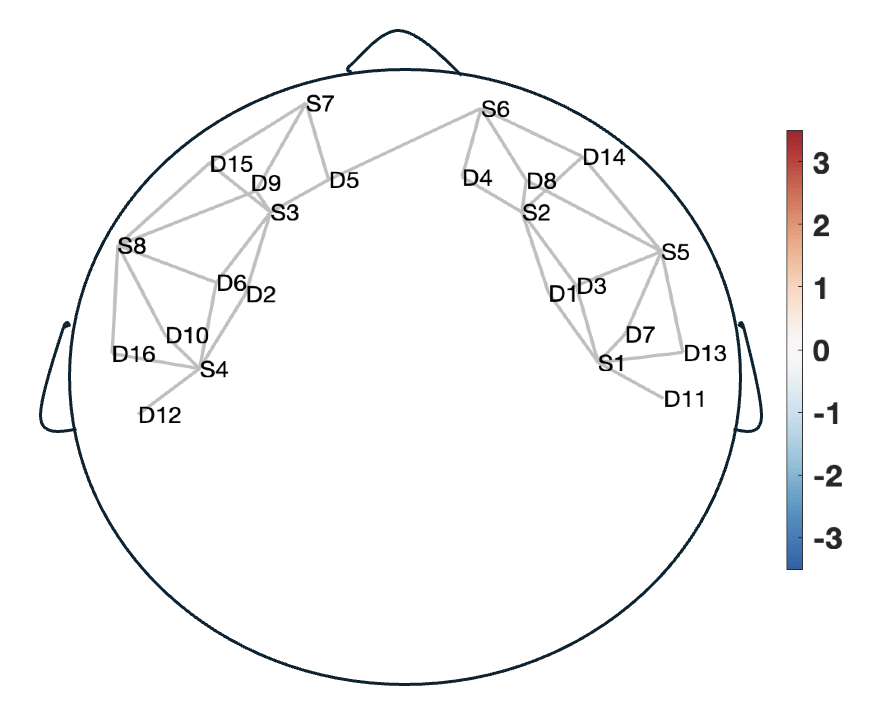**  b  **t-value** | 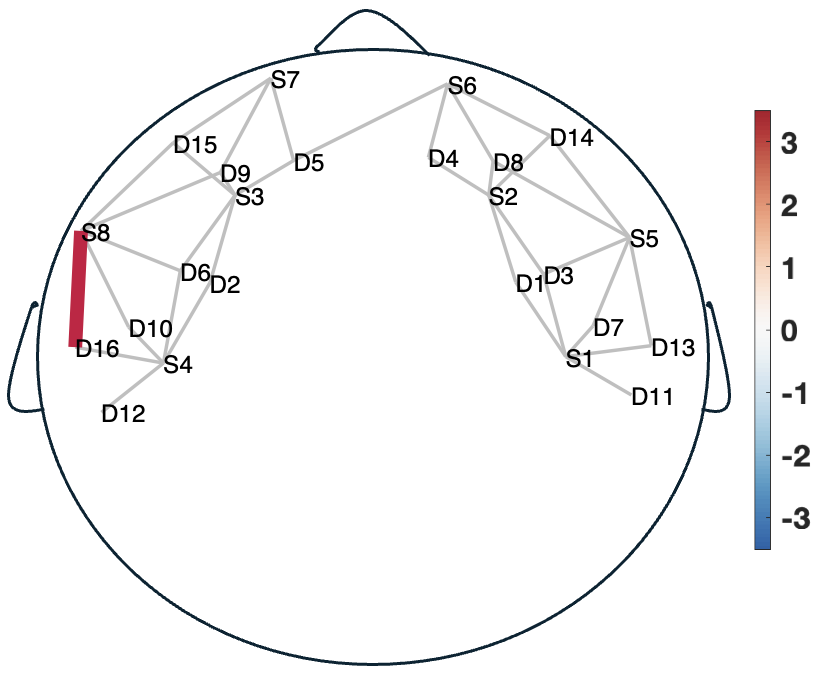  **t-value** |
| 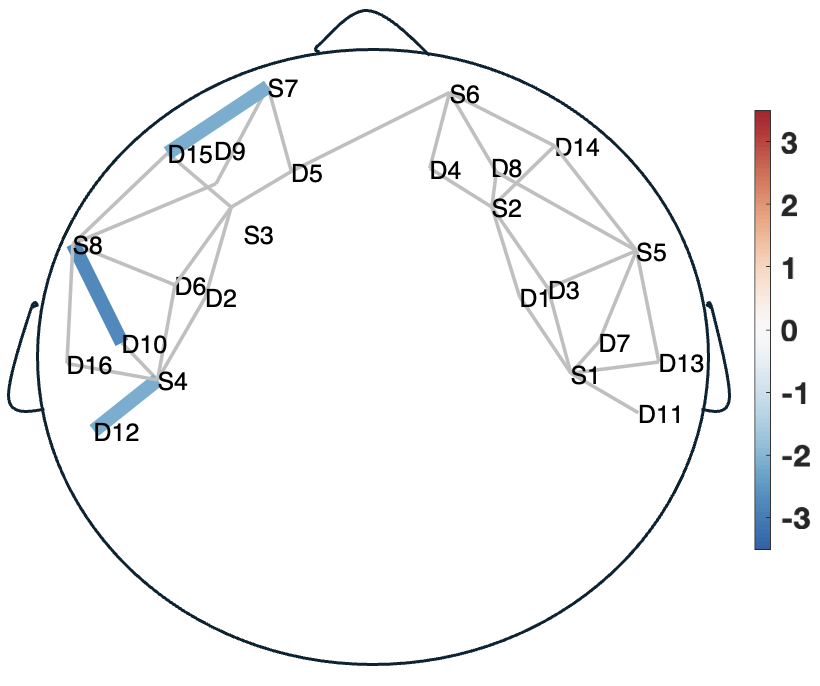  c  **t-value** | 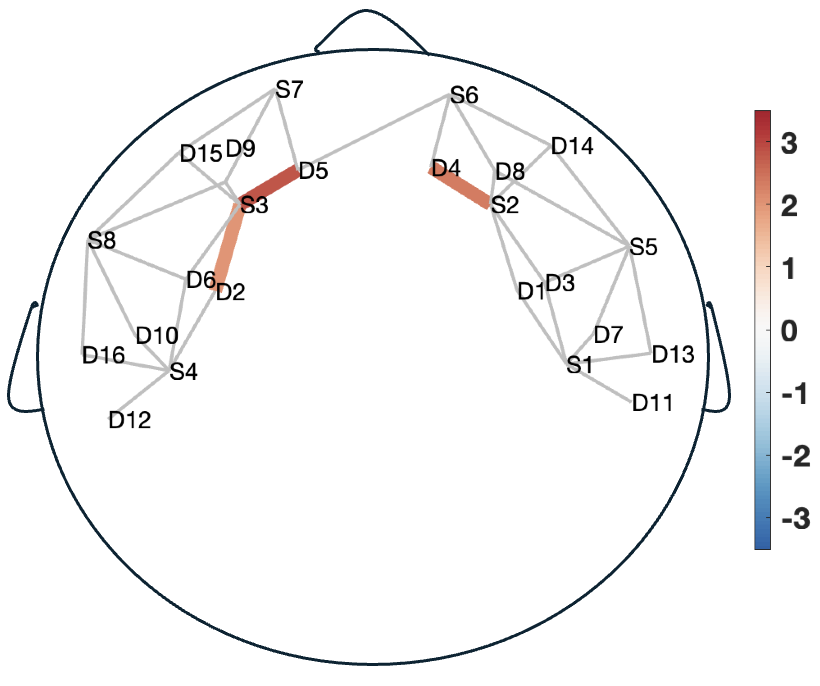  **t-value** |
| 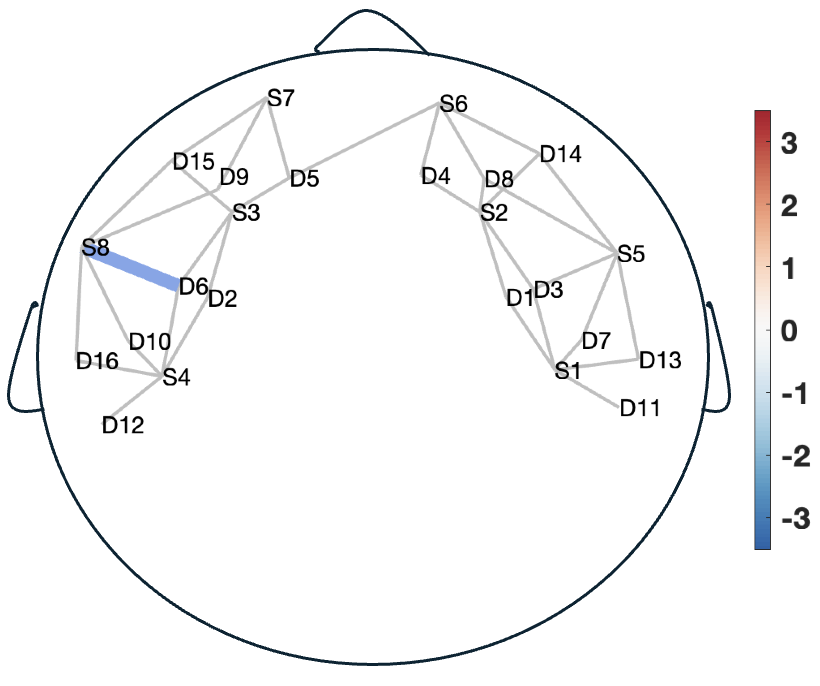  d | 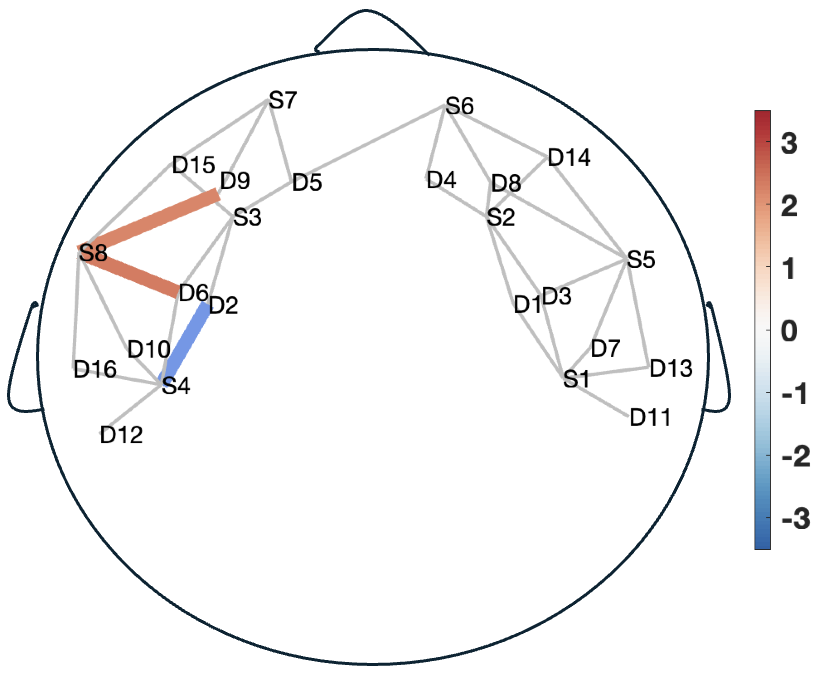  **t-value**  **t-value** |
| 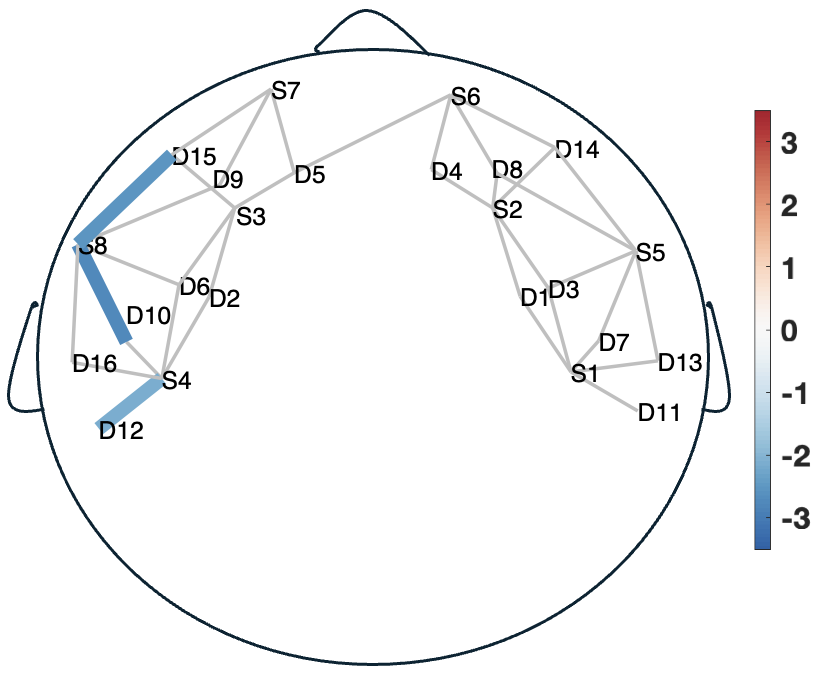  **t-value** | 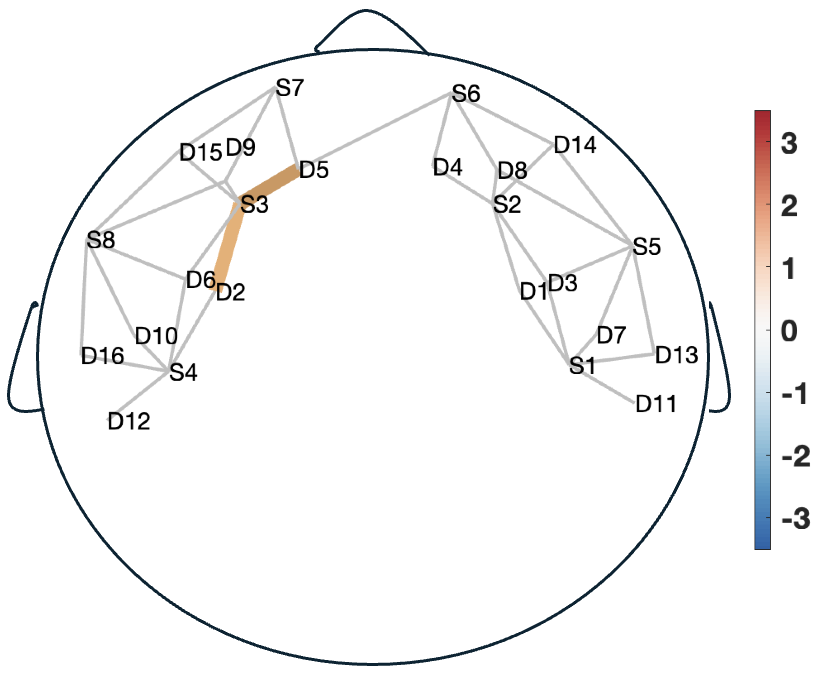  **t-value** |
| **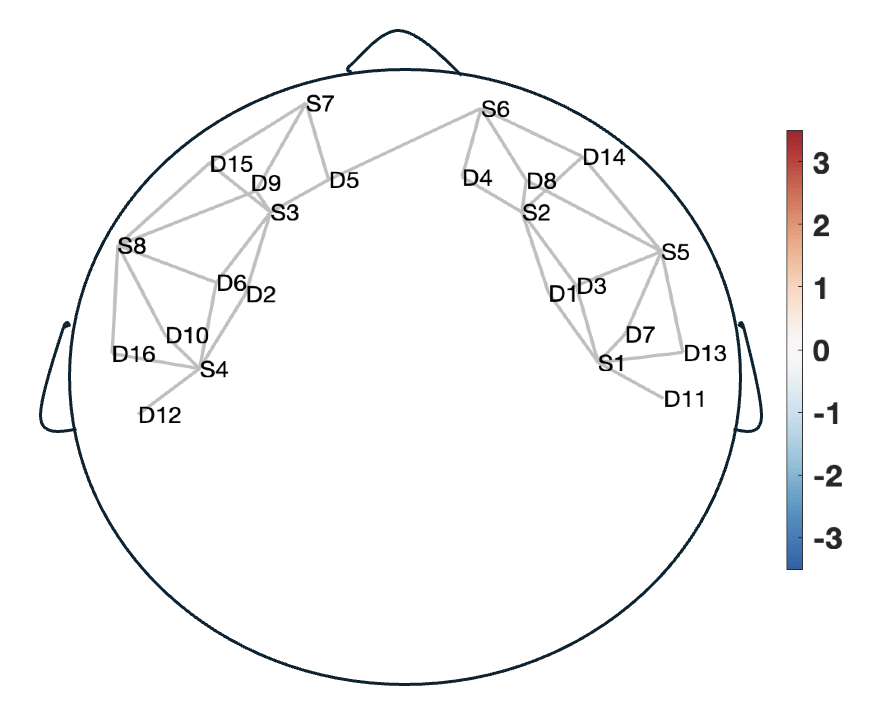**  **t-value** | 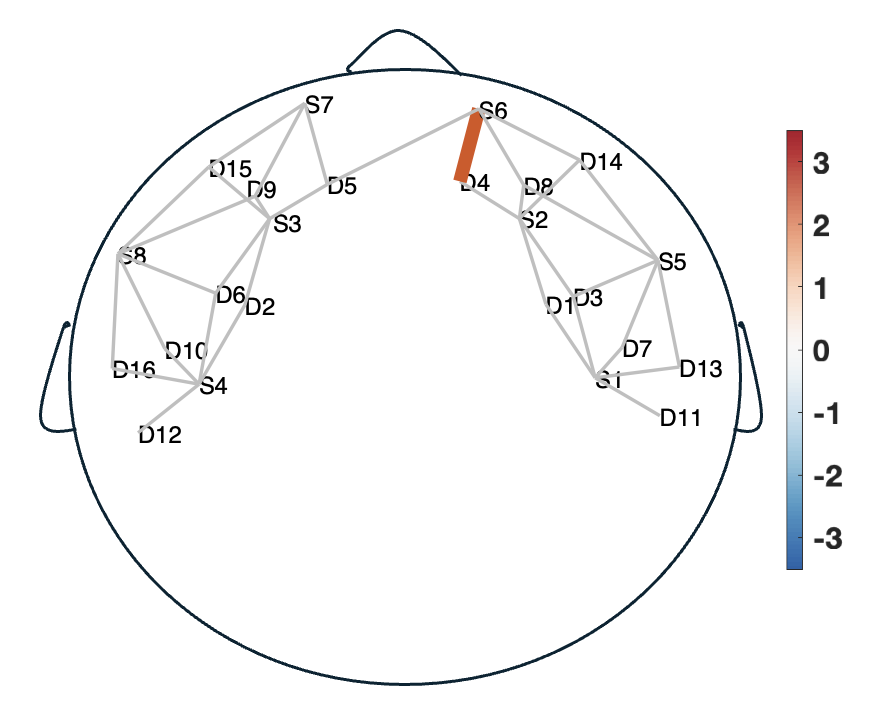 |

f

e

d

**t-value**

**Figure S5.** Channel maps of significant post–pre cortical activation (Oxy-Hb and deOxy-Hb) in the prefrontal cortex during the Go/No-go task conditions: (a) Go and (b) No-go. The color bar represents the t-values at each channel location across all participants.

b

a

| **deOxy-Hb** | **Oxy-Hb** |
| --- | --- |
| **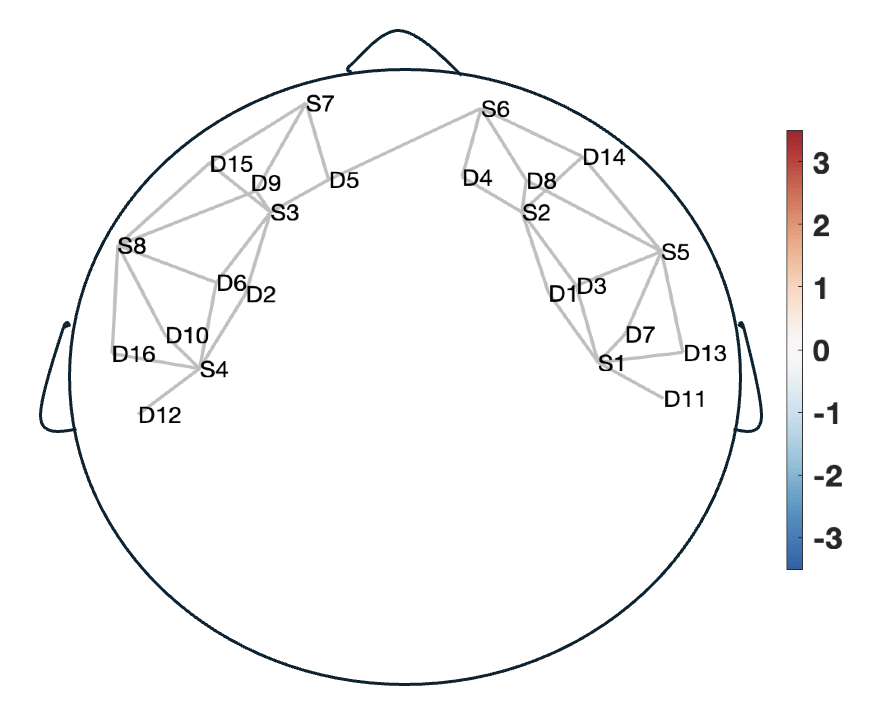** | **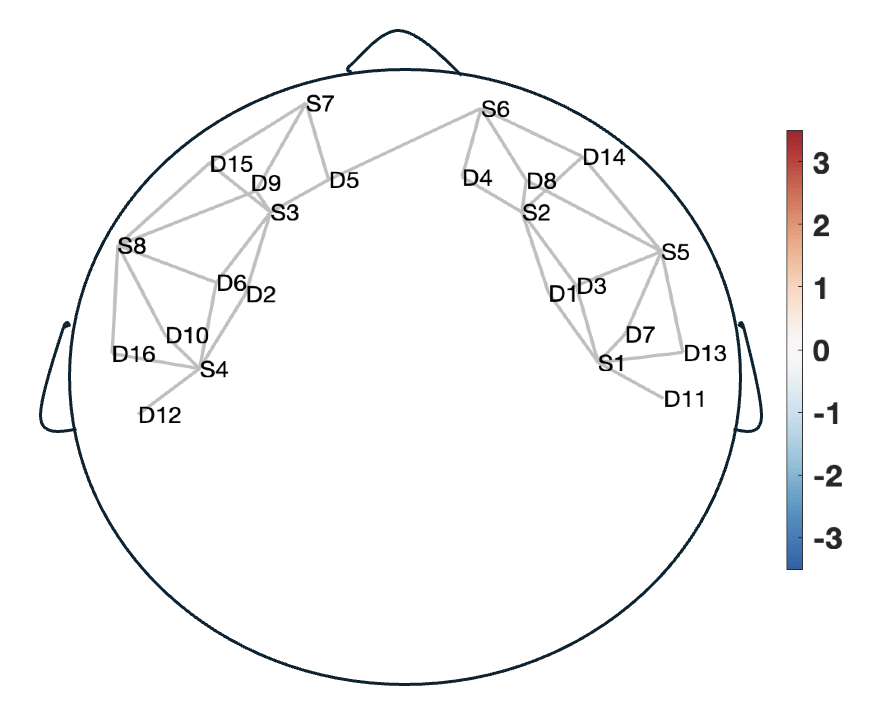**  **t-value** |
| **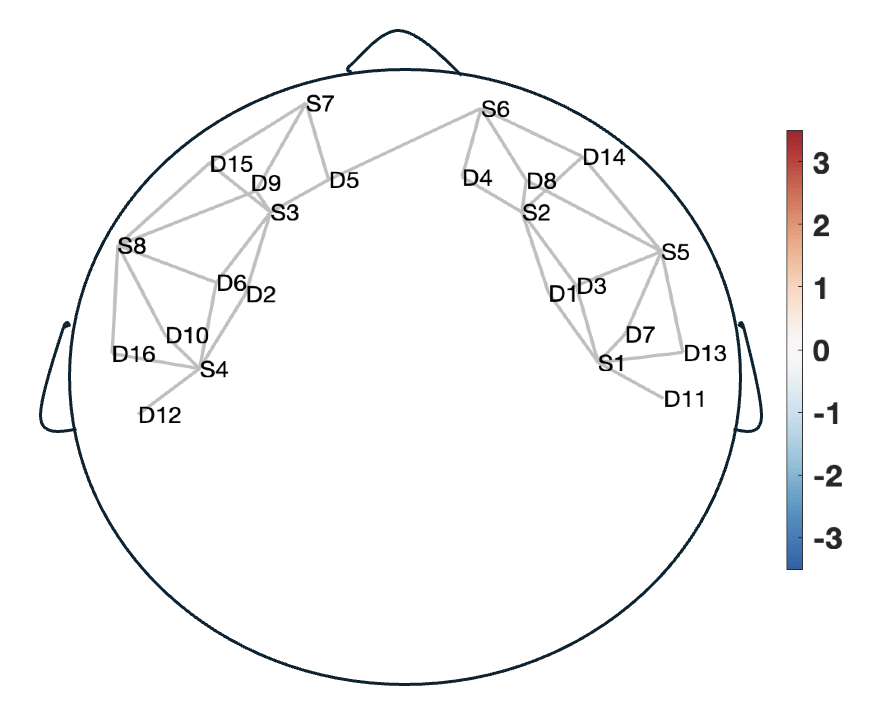**  **t-value** | **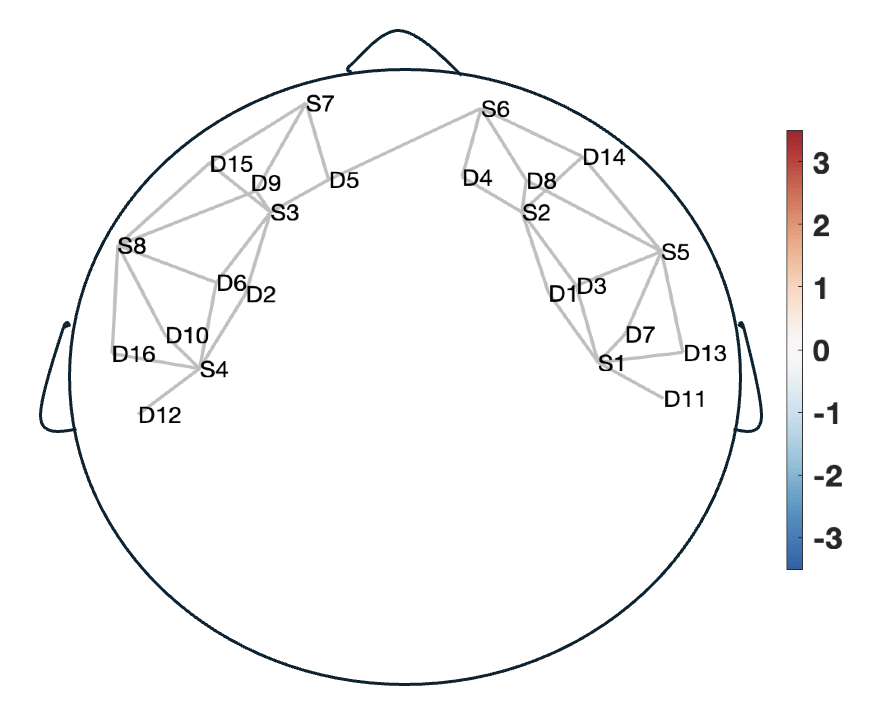**  **t-value**  **t-value** |

**Table S2.** MNI coordinates by channel and source-detector pairs, with estimated brain regions based on the AAL atlas.

| **Channel#** | **Source#** | **Detector#** | **MNI X** | **MNI Y** | **MNI Z** | **AAL area (Est.)** |
| --- | --- | --- | --- | --- | --- | --- |
| **1** | 1 | 1 | 80.4091 | 21.205 | 70.2417 | Dorsolateral PFC (R) |
| **2** | 1 | 3 | 88.3619 | 3.3474 | 64.8035 | Dorsolateral PFC (R) |
| **3** | 1 | 11 | 89.8454 | -13.5960 | 64.5663 | Dorsolateral PFC (R) |
| **4** | 1 | 13 | 88.7812 | -9.4398 | 55.2180 | Inferior Frontal Gyrus (R) |
| **5** | 2 | 1 | 63.9014 | 49.9305 | 49.2314 | Middle Frontal Gyrus (R) |
| **6** | 2 | 3 | 66.6794 | 46.4813 | 43.2807 | Middle Frontal Gyrus (R) |
| **7** | 2 | 4 | 41.9547 | 68.4834 | 25.6394 | Superior Frontal Gyrus (R) |
| **8** | 2 | 14 | 57.8031 | 49.7126 | 12.5706 | Superior Frontal Gyrus (R) |
| **9** | 3 | 2 | -52.9428 | 50.6653 | 57.5372 | Middle Frontal Gyrus (L) |
| **10** | 3 | 5 | -34.4210 | 68.8709 | 30.9296 | Superior Frontal Gyrus (L) |
| **11** | 3 | 6 | -56.7733 | 47.2818 | 50.9422 | Middle Frontal Gyrus (L) |
| **12** | 3 | 15 | -52.3534 | 50.0577 | 20.6902 | Superior Frontal Gyrus (L) |
| **13** | 4 | 2 | -66.8671 | 21.4644 | 82.6727 | Dorsolateral PFC (L) |
| **14** | 4 | 6 | -70.6976 | 18.0809 | 76.0777 | Dorsolateral PFC (L) |
| **15** | 4 | 12 | -77.7428 | -14.1437 | 79.5150 | Dorsolateral PFC (L) |
| **16** | 4 | 16 | -77.9138 | -9.3651 | 69.3578 | Inferior Frontal Gyrus (L) |
| **17** | 5 | 3 | 78.8554 | 18.3161 | 33.4819 | Inferior Frontal Gyrus (R) |
| **18** | 5 | 7 | 84.0332 | 3.9074 | 33.9945 | Inferior Frontal Gyrus (R) |
| **19** | 5 | 8 | 68.7417 | 30.0706 | 16.0520 | Middle Frontal Gyrus (R) |
| **20** | 5 | 13 | 84.4525 | -8.8797 | 24.4090 | Ventrolateral PFC (R) |
| **21** | 5 | 14 | 69.9821 | 21.5473 | 2.7718 | Orbitofrontal Cortex (R) |
| **22** | 6 | 4 | 27.2478 | 67.4585 | 0.0858 | Medial Frontal Gyrus (R) |
| **23** | 6 | 5 | 2.2562 | 67.5533 | 1.9412 | Medial Frontal Gyrus |
| **24** | 6 | 8 | 41.8558 | 57.2110 | 0.2971 | Medial Frontal Gyrus (R) |
| **25** | 6 | 14 | 43.0962 | 48.6877 | -12.9830 | Inferior Frontal Gyrus (Orbital, R) |
| **26** | 7 | 5 | -22.3681 | 68.4019 | 4.3152 | Medial Frontal Gyrus (L) |
| **27** | 7 | 9 | -36.3902 | 58.6583 | 5.9708 | Medial Frontal Gyrus (L) |
| **28** | 7 | 15 | -40.3006 | 49.5886 | -5.9242 | Inferior Frontal Gyrus (Orbital, L) |
| **29** | 8 | 6 | -70.5645 | 19.5719 | 43.1606 | Inferior Frontal Gyrus (L) |
| **30** | 8 | 9 | -62.2342 | 31.4174 | 24.8036 | Middle Frontal Gyrus (L) |
| **31** | 8 | 10 | -75.9978 | 4.0817 | 44.8844 | Inferior Frontal Gyrus (L) |
| **32** | 8 | 15 | -66.1446 | 25.2374 | 12.9086 | Orbitofrontal Cortex (L) |
| **33** | 8 | 16 | -77.7808 | -4.9845 | 36.4407 | Ventrolateral PFC (L) |

**Figure S6.** Natural setting of wearable fNIRS headband


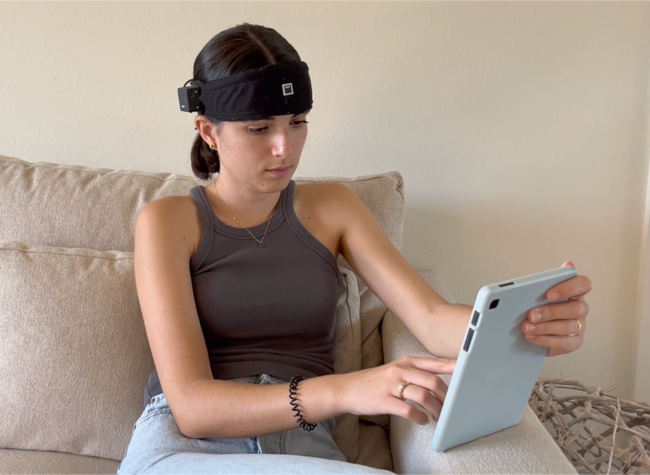

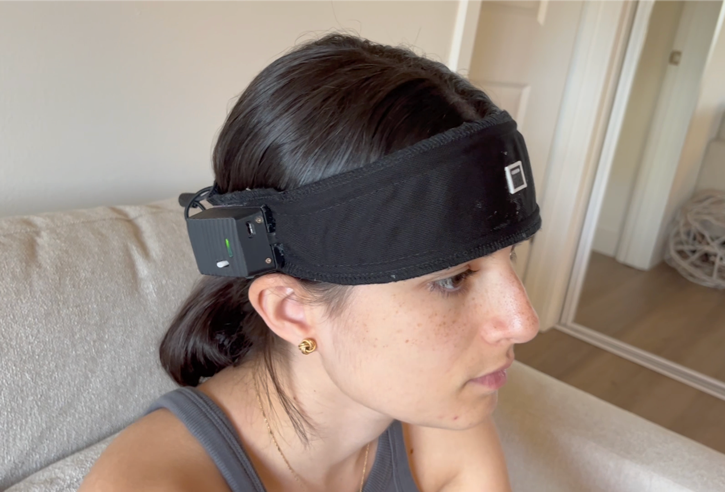

Supplement: Supplementary file 1 — Supplementary Information. [file 41598_2025_20844_MOESM1_ESM.docx]
